# mRNA Vaccines in Modern Immunotherapy for Non-Small Cell Lung Cancer (NSCLC)—A Comprehensive Literature Review with Focus on Current Clinical Trials

**DOI:** 10.3390/biomedicines13092187

**Published:** 2025-09-07

**Authors:** Jacek Kabut, Grzegorz J. Stępień, Tomasz Furgoł, Michał Miciak, Natalia Nafalska, Małgorzata Stopyra, Marcin Jezierzański, Krzysztof Feret, Iwona Gisterek-Grocholska

**Affiliations:** 1 Department of Oncology and Radiotherapy, Silesian Medical University, 40-514 Katowice, Poland; jkabut@sum.edu.pl (J.K.); igisterek@sum.edu.pl (I.G.-G.); 2 Faculty of Medicine, Medical University of Lodz, 90-419 Lodz, Poland; 3 Faculty of Medicine, Silesian Medical University, 41-800 Zabrze, Poland; s80882@365.sum.edu.pl (T.F.); s81185@365.sum.edu.pl (N.N.); s81569@365.sum.edu.pl (M.S.); s80961@365.sum.edu.pl (M.J.); 4 Faculty of Medicine, Wroclaw Medical University, 50-367 Wroclaw, Poland; michal.miciak@student.umw.edu.pl; 5 Faculty of Medicine, Academy of Silesia, 40-555 Katowice, Poland; krzysztof.feret@akademiaslaska.pl

**Keywords:** mRNA vaccine, lung cancer, non-small cell lung carcinoma, tumor antigens, immune response, immunotherapy, clinical oncology, molecular cancer therapy

## Abstract

Malignant neoplasms, like non-small cell lung cancer (NSCLC), remain a major global health challenge. Lung cancer is the leading cause of cancer-related deaths worldwide, with over two million new cases and 1.8 million deaths annually. NSCLC accounts for approximately 85% of cases, underscoring its substantial public health impact. Advances in molecular biology have driven the development of new therapies beyond traditional treatments. Among them, mRNA-based immunoadjuvant therapies, like cancer vaccines, have emerged as promising utilities in NSCLC by triggering targeted immune responses. The aim of this paper is to review ongoing and completed studies on mRNA vaccines in NSCLC. The efficacy of mRNA vaccines in NSCLC relies on the identification of immunogenic tumor-specific antigens, frequently derived from genomic profiling databases. Completed clinical trials have assessed the safety and potential benefit of selected mRNA vaccines—such as CV9202—administered alone or in combination with radiotherapy or tyrosine kinase inhibitors. Ongoing studies are exploring the therapeutic potential of mRNA-based approaches targeting defined molecular alterations in NSCLC, particularly in conjunction with Programmed Death-Ligand 1 (PD-L1) immune checkpoint inhibitors to enhance antitumor immune responses. mRNA vaccines have emerged as a promising therapeutic option for NSCLC, with the potential to enhance immune responses and limit tumor progression, as demonstrated in ongoing clinical trials. They offer the possibility of personalized treatment with relatively few side effects. However, larger and long-term studies are required to fully confirm their safety and efficacy. Future research should aim to identify the most effective antigens, enhance stability, and refine delivery strategies to improve efficacy and personalization, while also addressing immune suppression within the tumor microenvironment.

## 1. Introduction

Malignant neoplasms remain a major global health challenge due to their rising incidence and associated societal and economic burdens. Projections suggest over a million new cases annually by 2050, highlighting the urgent need for more effective, less toxic therapies. Advances in molecular biology, immunology, and biotechnology have significantly influenced clinical oncology, driving the development of novel therapeutic approaches beyond conventional treatments [1]. Among various malignancies, lung cancer represents a particularly pressing clinical concern, as it remains the leading cause of cancer-related deaths globally. In 2022, an estimated 2.5 million new cases of lung cancer were diagnosed, of which approximately 85% were classified as non-small cell lung cancer (NSCLC). Lung cancer accounted for 18.4% of all cancer-related deaths, corresponding to roughly 1.8 million fatalities, thereby remaining the leading cause of cancer mortality [2].

NSCLC, which encompasses adenocarcinoma, squamous cell carcinoma, and large cell carcinoma, arises predominantly from epithelial cells of the lung. Adenocarcinoma, the most common subtype, typically originates from type II alveolar cells. The pathogenesis of NSCLC is driven by a complex interplay of genetic, epigenetic, and environmental factors, with tobacco smoking accounting for approximately 85–90% of lung cancer cases. In addition to active smoking, exposure to second-hand smoke, familial predisposition, and contact with carcinogenic agents such as radon, asbestos, arsenic, chromium, beryllium, and nickel substantially increase the risk of disease. Other contributing factors include pulmonary fibrosis, HIV infection, and excessive alcohol consumption [3]. Key molecular alterations, including mutations in EGFR, KRAS, and ALK, drive oncogene activation or tumor suppressor gene inactivation, thereby promoting uncontrolled cell proliferation, angiogenesis, metastasis, and immune evasion. The tumor microenvironment, comprising cancer-associated fibroblasts and immune cells such as regulatory T cells and tumor-associated macrophages, further facilitates tumor progression by enhancing invasiveness and suppressing anti-tumor immune responses. Premalignant changes, including hyperplasia and metaplasia, may precede the development of malignancy [4].

Despite advancements in diagnostics and the introduction of targeted agents and immunotherapies, overall prognosis remains poor, with 5-year survival rates lingering below 20% in most populations. A significant proportion of patients exhibit primary or acquired resistance to current treatment modalities, underscoring the critical need for novel therapeutic strategies that are both more effective and well tolerated. While anti-PD-1/PD-L1 (programmed death receptor/ligand 1) immunotherapy and chemoimmunotherapy were pivotal advances in cancer treatment, current research has expanded toward more sophisticated strategies. These include chimeric antigen receptor T-cell therapy (CAR-T cell therapy), cancer vaccines, novel immune checkpoint inhibitors, oncolytic viruses, tumor-infiltrating lymphocytes (TILs), cancer-antigen specific T-cell receptors (TCRs) engineering, and multimodal immunotherapies [1,5].

One example of a further innovative approach is the development of therapies based on mRNA technology, which have gained recognition as potentially groundbreaking utilities in the treatment of various cancer types, including lung cancer. mRNA vaccines are now the focus of intensive research as a component of cancer therapy, capable of eliciting a specific immune response directed against tumor-associated antigens (TAAs). Various vaccine platforms are under investigation—such as cell-based, stem cell-based, peptide, microbial vector, nucleic acid (DNA/RNA), in situ, and exosome-based vaccines—delivered via routes including intravenous, intramuscular, subcutaneous, oral, or intra-dermal administration. Enhanced delivery methods, including gene vectors, electroporation, and nanotechnologies, are also being utilized to improve efficacy [5,6].

The aim of this paper is to present the current state of knowledge regarding the application of mRNA vaccines in NSCLC therapy, with particular emphasis on their efficacy, mechanism of action, and potential avenues for future development. The study is based on available literature, including the latest reports published up until 2025, which enables a comprehensive analysis of the issue and an evaluation of the future of this innovative therapeutic approach.

## 2. Landscape of mRNA Vaccine Research in NSCLC

A key factor in the efficacy of mRNA vaccines for NSCLC treatment is the identification of appropriate antigens capable of effectively enhancing the immune response. Current research is primarily focused on lung adenocarcinoma (LUAD), which is one of the most frequently diagnosed subtypes of this malignancy.

### 2.1. Antigen Identification and Selection

Intensive efforts are being made to utilize genetic databases for the identification of TAAs, suitable for mRNA vaccine development. However, the TAA-based approach faces limitations due to ectopic expression in healthy lung tissues, which can trigger on-target/off-tumor immune responses and severe autoimmune toxicity. Consequently, research focus has shifted toward neoantigen-based therapies as a promising alternative. In contrast, tumor-specific antigens (TSAs) are neoantigens arising from somatic mutations and are exclusively expressed in cancer cells, making them highly specific targets for immunotherapy. Neoantigen-targeted strategies include personalized cancer vaccines (e.g., EGFR T790M/C797S mutation neoantigen vaccine), adoptive cell therapies (e.g., TCR-T cells targeting the NY-ESO-1 antigen), neoantigen-specific antibody therapies (e.g., bispecific antibodies, BsAbs), and combination approaches [2,7]. An alternative strategy involves the use of mRNA encoding immunostimulatory molecules, designed to directly modulate the tumor microenvironment and enhance anti-tumor immune responses. One such candidate, mRNA-2752, encodes OX40L, IL-23, and IL-36γ and is currently under investigation in a dose-escalation study employing intratumoral administration in patients with advanced malignancies. Although results are not yet available, one of the dose-expansion cohorts includes patients with NSCLC who are either primary refractory or have developed secondary resistance to immune checkpoint blockade, underscoring the potential of this approach as a novel therapeutic avenue in NSCLC [8].

### 2.2. Design and Engineering of the mRNA Construct

Following antigen selection, the mRNA molecule is engineered to ensure stability, immunological safety, and efficient translation. To enhance protein expression in mRNA vaccines, the 5′ untranslated region (5′ UTR) is carefully designed to promote ribosome recruitment while minimizing elements that could impede translation, such as upstream open reading frames (ORFs) or regulatory motifs like iron-responsive elements. Approaches include using naturally efficient 5′ UTRs, such as those derived from human *β*-globin, or designing simplified synthetic sequences. Contemporary strategies increasingly employ machine learning techniques, including convolutional neural networks and generative models, which utilize experimental data to optimize 5′ UTR sequences for maximal protein expression while maintaining structural stability and regulatory function [9].

Codon optimization within the ORF is a critical aspect of mRNA design, involving the selection of codons that correspond to the host cell’s tRNA abundance to enhance translational efficiency. Tools such as the codon adaptation index (CAI) and codon stabilization coefficient (CSC) are commonly employed to guide these choices. Additionally, the secondary structure of mRNA must allow sufficient local flexibility to permit efficient ribosomal transit [10]. The 3′ untranslated region (3′ UTR) and poly(A) tail are also essential for mRNA stability and translation. The poly(A) tail interacts with poly(A)-binding proteins (PABPs) to protect mRNA from degradation and facilitate translation initiation. In vaccine design, sequences such as the *β*-globin 3′ UTR are frequently utilized due to their demonstrated ability to enhance protein expression. Selection of 3′ UTRs involves avoiding destabilizing elements, such as AU-rich regions or microRNA binding sites, while retaining regulatory motifs that support translation. Likewise, the poly(A) tail is typically optimized in length, often around 100–120 nucleotides, to ensure efficient ribosome recruitment and mRNA stabilization [11].

### 2.3. Delivery Systems

Efficient delivery of mRNA vaccines into target cells is a critical determinant of therapeutic efficacy, as it protects the mRNA from degradation by RNases and overcomes barriers associated with its large size, hydrophilic nature, and negative charge. Numerous delivery platforms have been explored, including lipid nanoparticles, lipoplexes, polyplexes, protamine complexes, gold nanoparticles, exosomes, cell-penetrating peptides, mesoporous silica nanoparticles, and various combinations [12,13]. These systems differ in their capacity to enhance mRNA stability, facilitate cellular uptake, and modulate immune responses. This review focuses on the application of delivery strategies, particularly in NSCLC, with peptide-based nanoparticles and cationic or ionizable lipid-based systems identified as the most promising approaches for effective mRNA delivery.

#### 2.3.1. Peptide-Assembled Systems

Two commonly used types of peptide-based nanoparticles are cell-penetrating peptides (CPPs) and protamines. Protamines are small, arginine-rich peptides that facilitate the condensation of mRNA into nanoparticles approximately 300 nm in size. In addition to their delivery function, protamines can stimulate the immune system via activation of Toll-like receptors 7 and 8 (TLR7 and TLR8). CPPs are short amino acid sequences capable of traversing cell membranes, primarily due to their positive charge, which promotes interaction with negatively charged membrane surfaces. Their amphipathic regions further enhance their ability to cross lipid bilayers, thereby improving membrane penetration and intracellular delivery of mRNA [14,15].

In the study by Mai et al., a cationic liposome–protamine complex (LPC) mRNA vaccine was evaluated via intranasal administration. The LPC formulation demonstrated enhanced cellular uptake and robust dendritic cell activation in vitro, eliciting a potent anti-tumor immune response. In vivo, intranasal immunization with LPC carrying mRNA encoding cytokeratin 19 significantly inhibited tumor growth in the aggressive Lewis lung cancer model, which serves as a murine analogue of human NSCLC. These findings highlight LPC as a safe and effective adjuvant, representing a promising platform for mRNA-based cancer vaccines [16].

#### 2.3.2. Lipid-Based Systems

**Figure 1:**
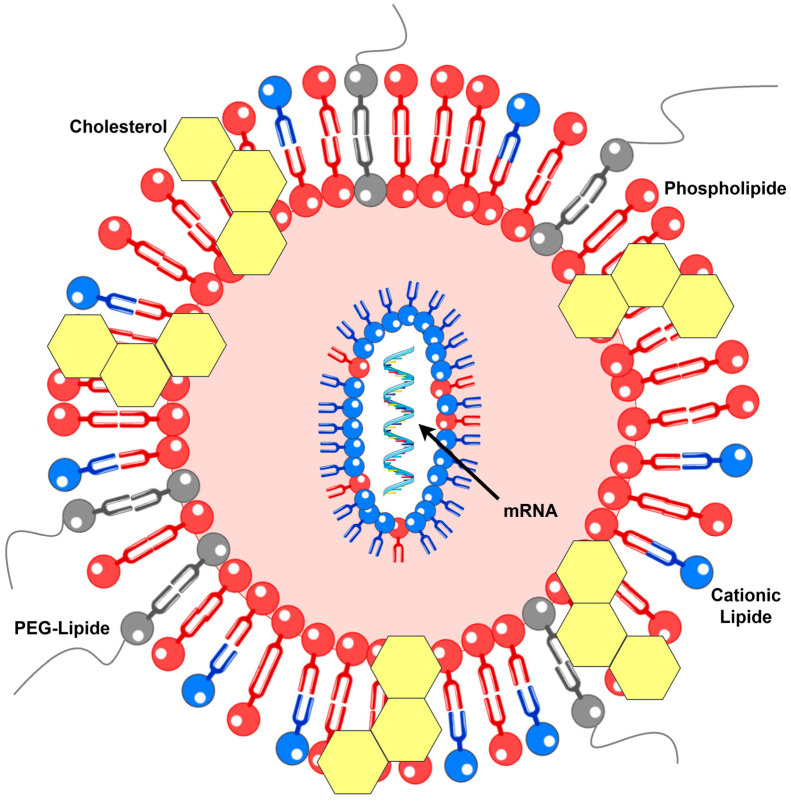
Molecular structure of the LNP (details in text). PEG—polyethylene glycol.

Lipid-based delivery systems, including lipoplexes and lipid nanoparticles (LNPs), represent the leading platforms for mRNA delivery. Although lipoplexes were initially developed for DNA and LNPs for siRNA, both systems have been successfully adapted for mRNA applications. Lipid-based mRNA vaccines typically comprise a cationic or ionizable lipid, a neutral phospholipid, a cholesterol derivative, and a stabilizing lipid, often a PEG-lipid conjugate, which prevents particle aggregation (Figure 1). Mixing the lipid solution with mRNA enables efficient encapsulation. Structurally, lipoplexes form multilayered assemblies, whereas LNPs possess dense, solid cores [13]. Examples of vaccines from this category discussed herein include mRNA-2752, as mentioned above, as well as mRNA-4157, CV9201, and CV9202 [17]. Notably, some sources describe CV9201 as a protamine-based mRNA vaccine; given the similarity, CV9202 may also be classified similarly, complicating the assignment of these formulations to distinct groups [13,18].

mRNA-4157/V940 is an mRNA vaccine encoding up to 34 neoantigens. It has been evaluated in combination with KEYTRUDA^®^ (pembrolizumab), an anti–PD-1 monoclonal antibody, in patients with stage III/IV melanoma at high risk of recurrence following complete surgical resection. The trial compared KEYTRUDA^®^ monotherapy with the combination of KEYTRUDA^®^ and mRNA-4157/V940, demonstrating that the combination reduced the risk of recurrence or death by 44% relative to monotherapy (HR = 0.56 [95% CI, 0.31–1.08]; one-sided *p* = 0.0266). An ongoing study is currently assessing mRNA-4157/V940 in combination with pembrolizumab versus placebo plus pembrolizumab in patients with completely resected stage II and IIIA/IIIB NSCLC [17,19,20,21].

CV9201 is a cancer immunotherapy based on RNActive^®^ technology, encoding five antigens associated with NSCLC: New York esophageal squamous cell carcinoma-1 (NY-ESO-1), melanoma antigen family C1 and C2 (MAGE-C1 and MAGE-C2), survivin, and trophoblast glycoprotein (5T4) [22]. CV9202 is a self-adjuvanting mRNA-based vaccine encoding six NSCLC-associated antigens: mucin 1 (MUC1), survivin, NY-ESO-1, 5T4 oncofetal antigen, and MAGE-C1 and MAGE-C2. Among these, NY-ESO-1 is highly immunogenic and associated with poor prognosis, while 5T4, survivin, and MUC1 are implicated in tumor progression and reduced survival. Notably, MUC1 has demonstrated clinical potential as a vaccine target in NSCLC trials [17,18,19,20,23].

### 2.4. Mechanism of Action

mRNA vaccines elicit both humoral and cytotoxic immune responses against NSCLC cells. These vaccines employ mRNA encapsulated within lipid nanoparticles (LNPs) to ensure efficient delivery to antigen-presenting cells (APCs), particularly dendritic cells (DCs). Following uptake through endocytosis, LNPs release the mRNA into the cytoplasm of APCs, where it undergoes translation into TAAs, thereby initiating antigen processing and presentation pathways that drive anticancer response [24].

In addition to uptake by professional APCs, LNPs from mRNA vaccines can also be internalized by non-APCs at the site of administration. Evidence indicates that somatic cells, such as muscle cells, fibroblasts, and keratinocytes, are capable of mRNA transfection and protein endocytosis. These cells may indirectly enhance immunity by transferring antigens to APCs and facilitating cross-presentation. Although the mechanisms remain incompletely understood, such interactions may significantly influence vaccine efficacy and safety. By contrast, in protein subunit vaccines, the overall amount of antigen internalized by non-APCs may represent a critical determinant of immune potency [25].

**Figure 2:**
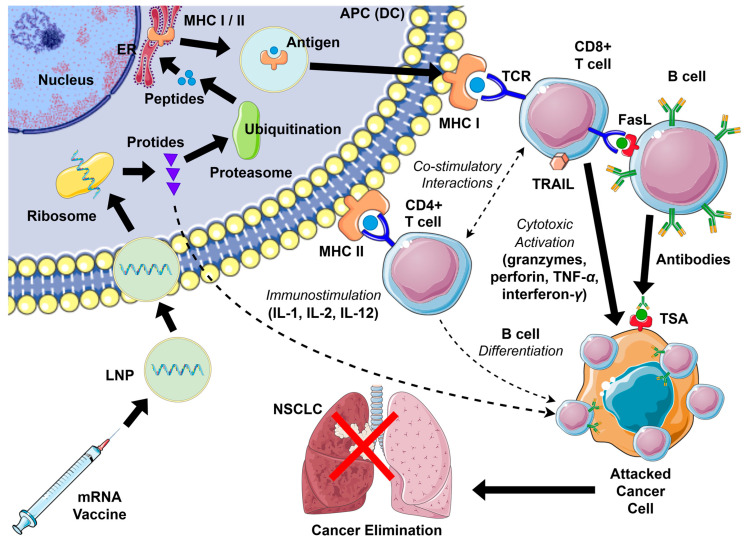
Activation scheme of the anti-cancer immune response with mRNA vaccines in NSCLC (details in text). APC—antigen-presenting cell; DC—dendritic cell; ER—endoplasmic reticulum; FasL—Fas ligand; IL—interleukin; LNP—lipid nanoparticle; MHC—major histocompatibility complex; NSCLC—non-small cell lung cancer; TCR—T-cell receptor; TNF—tumor necrosis factor; TRAIL—TNF-related apoptosis-inducing ligand; TSA—tumor-specific antigen.

Following cytoplasmic entry, mRNA is translated into proteins that contain neoantigens. After endosomal escape, ribosomes start to translate mRNA into protein, which is further processed by the ubiquitin–proteasome degradation system to produce peptides suitable for antigen presentation. Peptide fragments are transported via secretory vesicles to the endoplasmic reticulum and presented with MHC class I and II molecules on the APC surface, resulting in T-cell activation. Cytotoxic CD8^+^ T cells (Tc) engage MHC I, whereas exogenous proteins can be secreted, internalized by APCs, and presented on MHC II to activate helper CD4^+^ T cells (Th). T cells recognize antigens through their T-cell receptors (TCRs), forming an “immune synapse” via multiple interactions between immune checkpoints on APCs and lymphocytes. This response is further amplified by pro-inflammatory cytokines, including IL-1, IL-2, and IL-12, which promote T-cell proliferation and effector differentiation. CD8^+^ cells also secrete granzymes, perforin, TNF-*α*, and interferon-*γ*, mediating inflammation and inducing apoptosis in NSCLC cells. Other cytokines, such as Tumor Necrosis Factor-Related Apoptosis-Inducing Ligand (TRAIL) and Fas Ligand (FasL), also play a role in this process. Signaling through TRAIL and FasL helps maintain immune cell homeostasis, particularly among lymphoid populations, and serves as an effector mechanism by which cytotoxic cells eliminate damaged or malignant cells. Presented antigens can also activate B cells, which, with the assistance of CD4^+^ cells, differentiate into plasma cells that produce specific antibodies. This humoral response complements cytotoxic CD8^+^ T-cell activity, enhancing cancer cell elimination. Activation of co-stimulatory immune checkpoints further promotes the activation of antigen-specific T cells, while B lymphocytes are concurrently stimulated to generate anti-cancer antibodies [24,26,27,28,29]. An overview is provided in Figure 2.

Despite promising early results, evidence supporting mRNA vaccines in lung cancer remains limited, and further clinical trials are needed to confirm their therapeutic value. Ongoing studies are expected to clarify their potential in this challenging malignancy. An overview of available mRNA vaccines in NSCLC is summarized in Table 1.

## 3. Current Evidence from mRNA Vaccine Research in NSCLC

### 3.1. Clinical Studies Completed

In a phase I/II trial, Morse et al. evaluated an mRNA vaccine encoding carcinoembryonic antigen CEA delivered via dendritic cells in patients with metastatic CEA-positive tumors, including lung cancer. One lung cancer patient had a complete response, two partial responses, and three had stable disease. As lung cancer patients were excluded from phase II, further efficacy in this subgroup was not assessed [31].

In a phase I/IIa clinical study by Sebastian et al., the safety and efficacy of the mRNA vaccine CV9201 were evaluated in patients with advanced and metastatic NSCLC (stage IIIB or IV) following first-line therapy and disease stabilization (RECIST v1.0). A total of 46 patients received vaccine doses ranging from 400 to 1600 µg. The primary focus was on safety and T-cell immune response against the vaccine antigens. The vaccine was well tolerated at all dose levels, with 1600 µg selected for phase IIa. The most commonly observed adverse events included mild injection site reactions and flu-like symptoms. Eighteen patients showed at least a twofold increase in activated IgD^+^CD38^hi^ B cells. In phase IIa, disease stabilization was seen in 31% patients (9 of 29), while 69% patients experienced progression. Median PFS was 5.0 months, and median OS was 10.8 months. Two- and three-year survival rates were 26.7% and 20.7%, respectively [22].

A study by Papachristofilou et al. evaluated the safety and efficacy of the CV9202 mRNA vaccine combined with local radiotherapy as consolidation and maintenance therapy in 26 patients with stage IV NSCLC. Patients were stratified into three cohorts based on histology and prior treatments (chemotherapy or epidermal growth factor receptor tyrosine kinase inhibitors [EGFR-TKIs]). The vaccine was administered with 20 Gy of radiotherapy in four 5 Gy fractions. The regimen was well tolerated; common side effects included flu-like symptoms and injection site reactions. Enhanced immune responses were observed in 84% of patients, with 52% showing multi-antigen reactivity. Disease stabilization occurred in 46.2%, one patient achieved a partial response, and six showed >15% tumor shrinkage in non-irradiated lesions. Median PFS was 2.87 months, and OS was 13.95 months. The study supports further evaluation of CV9202, particularly in combination with immune checkpoint inhibitors [18].

The potential of combining mRNA vaccines with immune checkpoint inhibitors, such as PD-L1 and cytotoxic T-lymphocyte-associated protein 4 (CTLA-4) blockers, is also under investigation. In a non-randomized study, the efficacy and safety of the CV9202 vaccine administered in combination with durvalumab (anti-PD-L1 antibody) and tremelimumab (anti-CTLA-4 antibody) were evaluated in patients with NSCLC. A total of 61 patients were enrolled and divided into two groups: group A (n = 24) received the mRNA vaccine in combination with durvalumab, while group B (n = 37) received the vaccine alongside both durvalumab and tremelimumab. Among all participants, 57 patients received at least one dose of the investigational therapy. Treatment-related adverse events occurred in 56.5% of patients in group A and 55.9% in group B. In group A, partial response to treatment was achieved in 26.3% of patients, disease stabilization was observed in 36.8%, and progression occurred in 36.8%. In group B, these responses were 11.1%, 29.6%, and 59.3%, respectively. Due to the lack of randomization, these results should be interpreted with caution [32,33].

The ongoing Phase I trial LuCa-MERIT-1 (NCT05142189) is assessing the safety and efficacy of the BNT116 mRNA vaccine in NSCLC patients with PD-L1 expression > 50% who progressed after PD-1 inhibitor therapy. BNT116 encodes six NSCLC-associated antigens: melanoma antigen gene A3, A4, and C1 (MAGE A3, A4, and C1); claudin 6 (CLDN6); Kita-Kyushu lung cancer antigen-1 (KK-LC-1); and preferentially expressed antigen of melanoma (PRAME). The study evaluates BNT116 alone or in combination with cemiplimab, docetaxel, or an anti-CTLA-4 antibody, including its use in neoadjuvant and adjuvant settings. Preliminary results from 20 patients treated with BNT116 + cemiplimab showed a disease control rate of 80%, including 10% with partial response and 70% with stable disease. The median PFS was 5.5 months. Adverse events occurred in all patients, with 55% experiencing grade 3 and 25% grade 4 toxicity. The trial aims to enroll 160 patients, with completion expected in 2028 [34]. The summary of discussed completed studies is presented in Table 2.

### 3.2. Clinical Studies Underway

Although preliminary data support the safety and efficacy of mRNA vaccines in NSCLC, multiple clinical trials are ongoing to further explore their potential. Most studies are underway, with completion dates ranging from 2024 to 2035; however, the majority of findings remain unpublished.

The Phase I/II trial NCT02688686 evaluates the safety and efficiency of a DC-based vaccine encoding suppressor of cytokine signaling 1 (SOCS1), MUC-1, and survivin, in combination with cytokine-induced killer (CIK) cells, in patients with advanced NSCLC with bone metastases who received standardized treatment (n = 30). The primary outcomes of the study are complete response (CR) and partial response (PR) rates, assessed by RECIST criteria. The secondary outcome is the incidence of adverse events [35].

NCT03948763 assessed mRNA-5671/V941 targeting G12D, G12V, G13D, or G12C Kirsten rat sarcoma virus (KRAS gene) mutations in lung, colorectal, and pancreatic cancers (n = 70). There are no published results [36].

Another phase I trial, NCT05202561, is testing an RNA vaccine with or without PD-1 inhibition (navuliumab) in patients with KRAS-mutated (G12C, G12D, or G12V) solid tumors and specific HLA types (n = 10). Patients are divided into two study arms. Arm A focuses on the safety, efficacy, and pharmacokinetics of the vaccine. Patients receive a single intramuscular dose (each 600 ng) of the RNA vaccine on days 1, 4, 7, and 14. In Arm B, subjects of the study receive the RNA vaccine regime as in Arm A, along with maintaining therapy, intravenous injections of navuliumab (recommended dose: 3 mg/kg, every 2 weeks) starting on day 14. The primary outcome of this study is the incidence of adverse events. Secondary outcomes include antitumor activity (evaluated by RECIST and iRECIST criteria) and changes in immunoreactivity (peripheral lymphocyte T subtypes) [37].

Cevumeran (RO7198457), a personalized neoantigen vaccine, is under investigation in several trials. The phase 1a/1b NCT03289962 study (n = 272) was designed to evaluate the safety, tolerability, immune response, as well as pharmacokinetics of cevumeran, with or without atezolizumab (an anti-PD-L1 antibody) in patients with locally advanced or metastatic tumors [38,39]. The phase II study NCT04267237 aims to compare cevumeran + atezolizumab versus atezolizumab alone in stage II–III NSCLC patients who had positive circulating tumor DNA (ct-DNA) status after surgical resection and standard-of-care adjuvant platinum-doublet chemotherapy. The primary outcome of the study was disease-free survival; however, the study was withdrawn due to accrual timeline issues [40].

NCT03908671 investigates personalized neoantigen mRNA vaccines in patients with advanced esophageal cancer (stage IIIC [T4b-anyN-M0, anyT-N3-M0] or stage IV) or NSCLC (stage IIIB-IV) after the standard treatment failure or not suitable for standard treatment approaches. The primary objective of the study is to evaluate treatment-related adverse events [41].

Additional scheduled studies include NCT06685653 and NCT06735508, both evaluating personalized neoantigen mRNA vaccines with adebrelimab in resectable or recurrent/metastatic NSCLC [42,43]. NCT06077760 is a large phase III trial (n = 868) comparing intismeran autogene (V940) + pembrolizumab with placebo + pembrolizumab in patients with completely resected, margin-negative stage II–IIIB NSCLC. Intismeran is administered intramuscularly at a dose of 1 mg every 3 weeks for a total of 9 doses, combined with pembrolizumab. The primary outcome of the trial is disease-free survival, assessed up to 78 months [21]. Finally, NCT05557591 is comparing the combination of BNT116 with cemiplimab versus cemiplimab alone in first-line treatment of advanced NSCLC with PD-L1 ≥ 50% expression (n = 100). The effectiveness, PFS, OS, and adverse events will be evaluated [44]. The summary of discussed ongoing trials is presented in Table 3.

### 3.3. In Silico Studies

Bioinformatic analyses have enhanced the understanding of lung cancer immunogenicity and identified several antigens as potential targets for mRNA vaccine development. Zhao et al. highlighted that expression of bone morphogenetic protein 5 (BMP5) and claudin 5 (CLDN5) correlated positively with the infiltration of APCs, suggesting that higher levels of these genes may be linked to an enhanced immune response within the tissue microenvironment [45].

Other studies identified additional candidates, including G protein-regulated inducer of neurite outgrowth 1 (GPRIN1), myelin regulatory factor (MYRF), plexin B2 (PLXNB2), tripartite motif-containing protein 29 (TRIM29), ubiquitin-like modifier activating enzyme 6 (UBA6), and xanthine dehydrogenase (XDH) highlighted by Wang et al., as well as cyclin B1 (CCNB1), PCNA-associated factor (KIAA0101), PDZ-binding kinase (PBK), Opa interacting protein 5 (OIP5), and pleckstrin 2 (PLEK2) highlighted by Zhou et al., all linked to immune cell infiltration in LUAD [46,47]. Xu et al. proposed killer cell lectin-like receptor subfamily G member 1 (KLRG1) and core-binding factor, runt domain, alpha subunit 2; translocated to 3 (CBFA2T3), while Zhao et al. identified zinc finger CCCH-type containing 12D (ZC3H12D) and thioredoxin domain-containing protein 5 (TXNDC5) as further immunogenic targets [48,49]. In a murine model, an mRNA vaccine encoding MAGE-family member A1 (MAGE-A1) combined with a Toll-like receptor 4 (TLR4) agonist, monophosphoryl lipid A (mPLA), effectively activated an immune response and suppressed metastatic lung tumors, supporting its therapeutic potential [50].

## 4. Discussion

In recent years, the prognosis of patients with NSCLC has improved significantly, largely due to the integration of molecular diagnostics into clinical practice. The identification of oncogenic drivers such as EGFR and ALK enabled the use of targeted therapies, resulting in better outcomes [51]. For patients with metastatic NSCLC lacking actionable mutations, immune checkpoint inhibitors and chemotherapy remain standard treatments [52]. However, their effectiveness may be limited by side effects and resistance mechanisms [53,54,55]. To address these challenges, novel therapeutic strategies are under investigation. Among them, mRNA-based cancer vaccines have gained attention, especially following the rapid development and success of mRNA technologies during the COVID-19 pandemic, which accelerated their potential in oncology.

Anticancer mRNA vaccines are broadly classified into three groups based on the type of encoded component: TSAs, TAAs, or immune-regulatory molecules [56,57]. TSA-based vaccines encode cancer-specific neoantigens, while TAA-based formulations target genes expressed in tumors or germline tissues. These antigens, once presented to immune cells, can elicit targeted responses, offering a personalized approach with reduced toxicity [56,57,58]. In silico studies are key in identifying novel antigenic targets to improve efficacy. For instance, an mRNA vaccine encoding MAGE-A1 with mPLA activated innate and adaptive immunity and reduced bone metastases in a murine lung cancer model [50]. MAGE-A1 also showed oncogenic relevance in LUAD and may serve as a target for CAR-T cell therapy [59]. Ongoing identification and validation of such targets in large-scale trials is essential to advance TSA- and TAA-based immunotherapies in lung cancer.

Recent phase I/II trials have evaluated mRNA vaccines in NSCLC, focusing on safety, dosing, and early efficacy. Vaccines were tested alone or with chemotherapy, radiotherapy, or immune checkpoint inhibitors. While generally well tolerated and exhibiting a manageable safety profile, mRNA therapies have been associated with non-serious adverse events such as fatigue, fever, pneumonitis, and injection-site reactions (including erythema, pruritus, discoloration, and pain) [18,22,34]. More serious adverse events, such as pneumonitis, confusional state, pancreatitis, and systemic immune activation, were also observed [34,38,39]. Although adverse events did not occur among all the patients, and most commonly they are well tolerated, they should be considered when deciding on a suitable treatment approach. Early data suggest immune activation and disease control in subsets of patients [22,31,32,33,34].

In early trials, outcomes are modest but promising. For instance, CV9202 plus radio-therapy was associated with a median OS of 13.95 months and disease stabilization in 46.2% of patients [18]. BNT116 plus cemiplimab resulted in a median PFS of 5.5 months, with 80% disease control (70% with stable disease and 10% with partial responses) [34].

Differences in clinical outcomes across the referenced trials have been observed, with various delivery methods or mRNA platforms employed. One delivery strategy involves the use of dendritic cells. As reported by Morse et al., an mRNA vaccine encoding CEA was well tolerated in patients with CEA-expressing cancers. However, its clinical efficacy was rather limited, as 75% of patients (18/24) experienced a progressive disease during therapy [31]. Another approach utilizes mRNA formulated with protamine [18,22]. In the study investigating the CV9202 mRNA vaccine combined with radiotherapy, 46.2% of enrolled patients experienced a progressive disease [18]. Although the studies are not directly comparable, these observations may suggest that the choice of platform and delivery method may influence both safety and clinical effectiveness. Further comparative studies are required to identify and optimize the most effective delivery strategies to maximize immune responses and improve patient outcomes. LNPs are regarded as a next-generation delivery system and are being increasingly studied in clinical trials.

There is a growing recognition that biomarker-driven patient selection is becoming a major component in clinical trial design. Early studies, such as those by Morse et al., Sebastian et al., and Papachristofilou et al., were conducted without advanced molecular profiling, as they investigated tumor-associated antigens, e.g., CEA, NY-ESO-1, MAGE-C1, and MUC-1, typically overexpressed in tumors, but also detectable at low levels in some healthy tissue [18,22,31]. In the modern era of precision immunotherapy, targets for mRNA vaccines have become increasingly sophisticated. For instance, in the study by Lopez et al. (NCT03289962), at the stage of therapy design, next-generation sequencing (NGS) of tumor tissue was performed to identify somatic mutations. Based on bioinformatic analysis, individual patient-specific neoantigens were selected as targets, and individualized mRNA vaccines were then designed [38,39]. As described, the modern shift represents biomarker-driven patient selection, in contrast to broader cancer-type-driven selection.

KEY-NOTE-189 (metastatic non-squamous NSCLC) and KEYNOTE-407 (metastatic squamous NSCLC) reported median OS of 22.0 and 17.1 months, respectively, for chemoimmunotherapy [60,61]. In KEYNOTE-024, pembrolizumab alone yielded an OS of 26.3 months in patients with PD-L1 ≥ 50% [62].

Though outcomes with mRNA vaccines are currently inferior, these trials are early-phase and often involve heavily pretreated populations. Further studies are needed to explore their potential, especially in combination with checkpoint inhibitors.

As highlighted, mRNA cancer vaccines have shown encouraging early results in NSCLC and offer several advantages. Their design enables personalized therapy by targeting TSAs, potentially reducing toxicity and improving efficacy. They can activate both innate and adaptive immune responses and are generally well tolerated, with mostly mild side effects. Importantly, mRNA vaccines do not integrate into the host genome or carry a risk of infection, unlike DNA- or virus-based platforms [6,57,63,64,65,66].

Despite promising clinical outcomes, several challenges limit the full potential of mRNA vaccines and require further study. A key issue is mRNA instability and enzymatic degradation, which may impair delivery and immune activation. Strategies such as LNP encapsulation, nucleotide modification, and codon optimization address this problem [63,65,66,67,68]. Since conventional imaging may not adequately capture immune responses, alternative biomarkers like cytokine profiling, immune cell phenotyping, and ctDNA monitoring are proposed [57,67]. The optimal route of administration—intravenous versus intramuscular—also remains unresolved, with each having limitations [57,65,67]. Furthermore, tumor heterogeneity and the immunosuppressive microenvironment hinder antigen presentation and immune infiltration, posing additional obstacles [56,57,67,69].

## 5. Conclusions

mRNA vaccines have attracted significant attention in recent years and represent a promising addition to the treatment modalities for patients with NSCLC. Initial phase I and II clinical studies support their potential to activate the immune responses and reduce tumor burden, while offering a feasible and personalized treatment approach tailored to individual patients. However, with just a few early-phase clinical trials, it remains impossible to draw firm conclusions on factors influencing the safety and efficacy of mRNA vaccines in NSCLC. Key research gaps still consist of the optimal selection of targets (TSAs or TAAs) and delivery systems, as well as the durability of clinical responses. Another unresolved issue is the choice of effective therapeutic regimens, particularly combined with radiotherapy, chemotherapy, or immune checkpoint inhibitors. At present, mRNA vaccines cannot constitute standard oncologic care for NSCLC, but the initial research results strongly support their further development and clinical investigation.

## 6. Future Directions

The potential of mRNA technology in lung cancer treatment should be explored in randomized, multicenter phase II/III trials that prioritize large and diverse patient populations. Future work on mRNA vaccines should focus on rigorous assessment of long-term safety, as well as exploring the incidence and management of severe adverse events. Comparative studies on delivery strategies and administration routes are essential to optimize efficiency. Another potential direction is the integration of NGS-based tumor profiling into the routine design of mRNA vaccines, which constitutes a novel approach to modern immunotherapy, therefore enhancing vaccine specificity. To overcome immune suppression in the tumor microenvironment, combination strategies—such as immune checkpoint inhibitors or CRISPR-Cas9 modulation—may offer synergistic benefits [57,67,69]. Finally, detailed profiling of vaccine-induced immune responses is essential to optimize outcomes.

## Figures and Tables

**Table 1:** Summary of available mRNA vaccines in NSCLC [8,17,30].

Vaccine Name	Delivery System	Main Mode of Action	Encoding Antigens	Administration Route
CV9201	LNP/protamine	T-cell stimulation	NY-ESO-1, MAGE-C1, MAGE-C2, survivin, 5T4	Intradermal
CV9202	LNP/protamine	T-cell stimulation	NY-ESO-1, MAGE-C1, MAGE-C2, survivin, 5T4, MUC-1	Intradermal
BNT116	Lipoplex	T-cell stimulation	MAGE-A3, MAGE-A4, MAGE-C1, CLDN6, KK-LC-1, PRAME	Intravenous
mRNA-2752	LNP	T-cell stimulation, pro-inflammatory cytokines induction	OX40L, IL-23, IL-36γ	Intratumoral
mRNA-4157/V940	LNP	T-cell stimulation, inducing de novo T-cell responses	Personalized antigens (up to 34)	Intramuscular
mRNA-5671/V941	LNP	T-cell stimulation	KRAS: G12D, G12V, G13D, G12C	Intramuscular

Annotation. LNP—lipid nanoparticle; NY-ESO-1—New York esophageal squamous cell carcinoma-1; MAGE—melanoma antigen family; 5T4—trophoblast glycoprotein; MUC-1—mucin 1; KRAS—Kirsten rat sarcoma virus; IL—interleukin.

**Table 2:** Summary of completed clinical studies regarding mRNA vaccines for NSCLC [18,22,31,32,34].

Study Name and Type	Vaccine Name	Vaccine Targets	No. of Enrolled Patients	Intervention	Key Findings
Morse et al. (Phase I/II)	CEA mRNA Vaccine	CEA	N = 29 (Phase I) N = 13 (Phase II)	CEA mRNA Vaccine	Phase I—18/24 evaluable patients had progression (75%) Phase II—median follow-up: 429 days, recurrence: 9/13 (69.2%) Median time to recurrence: 122 days; 3/13 remained NED > 500 days (23.1%); 3/13 died (23.1%)
Sebastian et al. (Phase I/IIa)	CV9201	NY-ESO-1, MAGE-C1, MAGE-C2, survivin, 5T4	N = 46	CV9201	Median PFS: 5.0 months (95% CI: 1.8–6.3); 6-month PFS: 38.9%; 12-month PFS: 16.7% Median OS: 10.8 months (95% CI: 8.1–16.7); 1-year OS: 44.4%; 2-year OS: 26.7%; 3-year OS: 20.7%
Papachristofilou et al. (Phase Ib)	CV9202 (BI 1361849)	NY-ESO-1, MAGE-C1, MAGE-C2, survivin, 5T4, MUC-1	N = 26	Stratum 1: CV9202 + local radiation + pemetrexed Stratum 2: CV9202 + local radiation Stratum 3: CV9202 + local radiation + gefitinib/erlotynib	Grade ≥ 3 CV9202- and/or radiation-related AEs: 15.4% No serious CV9202- related TEAEs SD as best response: 46.2% Median PFS: 2.87 months (95% CI: 1.43–4.27) Median OS: 13.95 months (95% CI: 8.93–20.87)
Gandhi et al. (Phase I/II)	CV9202 (BI 1361849)	NY-ESO-1, MAGE-C1, MAGE-C2, survivin, 5T4, MUC-1	N = 61	Arm A: CV9202 + durvalumab Arm B: CV9202 + durvalumab + tremelimumab	TRAEs: 56.5% (Arm A), 55.9% (Arm B) SAEs: 4.3% (Arm A), 8.8% (Arm B) PR: 26.3% (Arm A), 11.1% (Arm B) SD: 36.8% (Arm A), 29.6% (Arm B) PD: 36.8% (Arm A), 59.3% (Arm B)
Atmaca et al. (Phase I)	BNT116	MAGE-A3, CLDN6, KK-LC-1, PRAME, MAGE-A4, MAGE-C1	N = 20	BNT116 + cemiplimab	TEAEs: 100% (45% grade 1/2; 55% grade 3) PR: 10% SD: 70% Median PFS: 5.5 months (95% CI: 2.9–9.5)

Annotation. NED—no evidence of disease; PFS—progression-free survival; OS—overall survival; AE—adverse event; TEAE—treatment-emergent adverse event; SD—stable disease; TRAE—treatment-related adverse event; SAE—serious adverse events; PR—partial response; PD—progressive disease.

**Table 3:** Summary of clinical studies underway regarding mRNA vaccines for NSCLC [21,35,36,37,38,40,41,42,43,44].

Trial Name and Type	Aim	Vaccine Targets	Key Findings	Completion
NCT02688686 (Phase I/II)	Evaluation of safety and efficacy of vaccine in combination with DCs and CIKs in patients with advanced NSCLC with bone metastasis	SOCS1, MUC-1, survivin	No published results (Unknown status)	–
NCT03948763 (Phase I)	Evaluation of safety and tolerability of mRNA-5671/V941 in monotherapy and in combination with pembrolizumab in patients with KRAS-mutated cancers	KRAS: G12D, G12V, G13D, G12C	No published results (Terminated)	–
NCT05202561 (Phase I)	Evaluation of safety and efficacy of RNA vaccine as monotherapy or in combination with PD-1 inhibitor in patients with KRAS mutation	KRAS: G12D, G12C, G12V	No published results (Unknown status)	January 2024
NCT03289962 (Phase Ia/Ib)	Evaluation of the safety, tolerability, immune response, and pharmacokinetics of cevumeran (RO7198457) in monotherapy and with atezolizumab in patients with NSCLC	Personalized neoantigens	Poly-epitopic neoantigen-specific CD4^+^/CD8^+^ T-cell responses in 71% of patients, detectable up to 23 months after initiation Autogene cevumeran-induced T cells constitute up to 7.2% of tumor-infiltrating T cells Clinical activity observed	March 2025
NCT04267237 (Phase I)	Evaluation of efficacy, safety, and pharmacokinetics of cevumeran (RO7198457) in combination with atezolizumab in patients with stage II–III NSCLC after surgery and chemotherapy	Personalized neoantigens	No published results (Withdrawn)	September 2025
NCT03908671 (Phase I/II)	Evaluation of safety, tolerability, and efficacy of mRNA vaccines encoding neoantigens in patients with esophageal cancer and NSCLC after treatment failure	Personalized neoantigens	No published results (Recruiting)	December 2025
NCT06685653 (Phase II)	Evaluation of safety and tolerability of personalized mRNA vaccine RGL-270 + adebrelimab in patients with operable NSCLC and recurrence after first-line treatment	Personalized neoantigens	No published results (Not yet recruiting)	November 2026
NCT06735508 (Phase I/II)	Evaluation of safety, tolerability, immunogenicity, and efficacy of personalized mRNA neoantigen vaccine with adebelimab in patients with NSCLC	Personalized neoantigens	No published results (Not yet recruiting)	December 2026
NCT06077760 (Phase III)	Comparison of efficacy of V940 + pembrolizumab vs. placebo + pembrolizumab in patients with completely resected stage II and IIIA/IIIB NSCLC	Personalized neoantigens	No published results (Recruiting)	December 2035
NCT05557591 (Phase I/II)	Evaluation of safety, tolerability, and efficacy of BNT116 + cemiplimab in patients with advanced NSCLC	Fixed combination of shared TSAs (MAGE-A3, MAGE-A4, MAGE-C1, CLDN6, KK-LC-1, PRAME)	No published results (Active, not recruiting)	June 2027

Annotation. CIK—cytokine-induced killer; CLDN—claudin; DC—dendritic cell; KK-LC—Kita-Kyushu lung cancer antigen; KRAS—Kirsten rat sarcoma virus; MAGE—melanoma-associated antigen; MUC—mucin; NSCLC—non-small cell lung cancer; PRAME—preferentially expressed antigen of melanoma; SOCS—suppressor of cytokine signaling; TSA—tumor-specific antigen.

## Data Availability

The datasets used and/or analyzed during the current study are available from the corresponding author upon reasonable request.
